# Development of an at‐home cognitive “vital sign” to detect cognitive changes in Alzheimer's disease

**DOI:** 10.1002/alz70857_106935

**Published:** 2025-12-25

**Authors:** Meaghan AM McKenna, Charlotte B Curnin, John Torous, Maurice Smith, Daniel Z. Press

**Affiliations:** ^1^ Beth Israel Deaconess Medical Center, Boston, MA, USA; ^2^ Harvard University, Boston, MA, USA; ^3^ Harvard University School of Enginering and Applied Sciences, Cambridge, MA, USA; ^4^ Department of Neurology, Harvard Medical School, Boston, MA, USA

## Abstract

**Background:**

Patients with Early Alzheimer's Disease (AD) can have fluctuations in their cognitive abilities. A rapid, significant change could indicate a serious health condition ranging from a urinary tract infection to delirium from brain edema due to an amyloid‐reducing therapy (ARIA). Unfortunately, there are no clinical tools currently in use to frequently assess cognition at home. Such a tool could not only detect acute changes from delirium, but also gradual changes related to disease progression. This study aims to investigate the utility, convenience, and feasibility of long‐term use of a novel online cognitive assessment (D‐Cog) in detecting cognitive changes at home.

**Method:**

We have designed a simple cognitive test, the D‐Cog, in the form of a “cognitive vital sign” that can be performed regularly at home in under 2 minutes. Pilot data on the utility of the D‐Cog was collected from 82 patients (mean age 74, 54 female), clinically diagnosed with MCI or AD by clinicians in the Beth Israel Deaconess Medical Center (BIDMC) Cognitive Neurology Unit (CNU). In addition, 15 participants with MCI or AD will be recruited to complete the D‐Cog daily for 6 months at home with study staff monitoring the fluctuations in data remotely.

**Result:**

28 participants were unable to complete the task following a demonstration and/or prompting from study staff and were subsequently excluded. The 54 included participants (mean age 76, 31 female) averaged a D‐Cog score of 3.9 and a MMSE score of 22. D‐Cog and MMSE scores were found to be highly correlated (*r* = 0.56, *p* =  1.3e‐5). In anecdotal reports following D‐Cog administration, patients indicate the task would be easy for them to complete at home.

**Conclusion:**

The D‐Cog assessment is feasible in clinical practice for AD patients, and patients would be willing to complete the D‐Cog remotely while at home. D‐Cog scores compare well against standardized pen‐and‐paper neuropsychological scores but offer the convenience of at‐home use and more frequent self‐administration. Future work investigating the utility and convenience of the D‐Cog as a daily at‐home assessment is promising as an accurate measure of cognitive ability, potentially offering early detection of delirium or ARIA.

